# Associations among Cognitive Functions, Plasma DNA, and White Matter Integrity in Patients with Early-Onset Parkinson's Disease

**DOI:** 10.3389/fnins.2017.00009

**Published:** 2017-01-24

**Authors:** Yueh-Sheng Chen, Meng-Hsiang Chen, Cheng-Hsien Lu, Pei-Chin Chen, Hsiu-Ling Chen, I-Hsiao Yang, Nai-Wen Tsai, Wei-Che Lin

**Affiliations:** ^1^Department of Diagnostic Radiology, Kaohsiung Chang Gung Memorial Hospital, Chang Gung University College of MedicineKaohsiung, Taiwan; ^2^Department of Neurology, Kaohsiung Chang Gung Memorial Hospital, Chang Gung University College of MedicineKaohsiung, Taiwan; ^3^Department of Biological Science, National Sun Yat-Sen UniversityKaohsiung, Taiwan; ^4^Department of Biomedical Imaging and Radiological Sciences, National Yang-Ming UniversityTaipei, Taiwan

**Keywords:** cognitive impairment, diffusion tensor imaging, early-onset Parkinson's disease, white matter, plasma DNA

## Abstract

Early-onset Parkinson's disease (EOPD) patients are symptomatic at a relatively young age, and the impacts of the disease on both the patients and their caregivers are dramatic. Few studies have reported on the cognitive impairments seen in EOPD, and the results of these studies have been diverse. Furthermore, it is still unclear what microstructural white matter (WM) changes are present in EOPD patients. As such, we conducted this study to investigate the microstructural WM changes experienced by EOPD patients and their association with cognitive function and plasma DNA levels. We enrolled 24 EOPD patients and 33 sex- and age-matched healthy volunteers who underwent complete neuro-psychological testing (NPT) to evaluate their cognitive function and diffusion tensor imaging (DTI) scanning to determine their fiber integrity. The plasma DNA measurements included measurements of nuclear and mitochondrial DNA levels. Fractional anisotropy (FA) maps were compared using voxel-based statistics to determine differences between the two groups. The differences in DTI indices and NPT scores were correlated after adjusting for age, sex, and education. Our results demonstrate that patients with EOPD have elevated nuclear DNA levels and wide spectrums of impairments in NPT, especially in the executive function and visuospatial function domains. Exploratory group-wise comparisons of the DTI indices revealed that the patients with EOPD exhibited lower DTI parameters in several brain locations. These poorer DTI parameters were associated with worse cognitive performances and elevated plasma nuclear DNA levels, especially in the anterior thalamic radiation region. Our findings suggest that the thalamus and its adjacent anterior thalamic radiation may be important in the pathogenesis of EOPD, as they appear to become involved in the disease process at an early stage.

## Introduction

Parkinson's disease (PD) is one of the most common neurodegenerative diseases worldwide (Aarsland et al., 2009). In addition to its characteristic motor symptoms, PD is associated with a spectrum of cognitive dysfunction, ranging from mild cognitive impairment to dementia (Kehagia et al., 2010). Although PD mainly affects older patients, with a mean age of onset at around 60 years old, some patients develop it at a relatively early age (Schrag et al., 2003). Since these younger patients are often the wage earners in their families, the cognitive impairment caused by PD may cause greater impacts on them and their families than does the cognitive impairment afflicting older onset PD patients. Studies have shown that the cognitive impairments in PD occur within various domains, including the attention, executive, memory, language, and visuospatial domains (Litvan et al., 2012). However, the cognitive function of patients with early-onset PD (EOPD) is less well-studied, with only a few studies having reported that EOPD patients may experience slower disease progression and less overall cognitive impairment relative to other PD patients (Hietanen and Teräväinen, 1988; Wickremaratchi et al., 2009). The underlying pathophysiology for the cognitive impairments seen in EOPD patients is also yet to be determined.

Diffusion tensor imaging (DTI) is an advanced magnetic resonance imaging (MRI) technique that has been used to evaluate the brains of patients affected by various clinical conditions, including brain tumors, hepatic encephalopathy, psychiatric disorders, epilepsy, and neurodegenerative disorders (Chaudhary and Duncan, 2014; Lerner et al., 2014; Lin et al., 2014a; Teipel et al., 2014; Arat et al., 2015). Studies using DTI have demonstrated extensive microstructural white matter (WM) damage in PD patients (Karagulle Kendi et al., 2008; Agosta et al., 2013; Kim et al., 2013). Recent studies have further established clear associations between the cognitive impairments of PD patients and their WM microstructural changes (Gallagher et al., 2013; Theilmann et al., 2013; Zheng et al., 2014). DTI can be used to assess WM microstructural integrity by measuring the water diffusion characteristics in tissues (Le Bihan, 2003). As such, analyzing the WM microstructural changes in EOPD patients may help us understand the pathology of the disease and further delineate its relationship with the resulting cognitive impairments.

There is growing evidence that oxidative stress and neuroinflammation play important roles in the pathogenesis of PD (More et al., 2013; Tolleson and Fang, 2013; Coppedè and Migliore, 2015). These insults may then cause neuronal cell death in the brain (Taylor et al., 2013). Neuron loss in PD is due to both apoptosis and necrosis (Perier et al., 2012). These processes, together with the disruption of the blood-brain barrier, may in turn cause free plasma DNA to be detected in peripheral blood (Martin et al., 1998; Cabezas et al., 2014). Elevated levels of plasma DNA have been shown to be associated with cell death in various diseases, including various cancers, traumas, infections, and vascular events (Wu et al., 2002; Lam et al., 2003; Rainer et al., 2003; Rhodes et al., 2006; Lu et al., 2010; Arnalich et al., 2013). However, the presentation and role of plasma DNA in EOPD patients remains unclear.

The present study aimed to investigate the WM microstructural integrities, cognitive statuses, and plasma DNA levels of EOPD patients, as well as to further explore the associations among them. First, the cognitive statuses, WM microstructural integrities, and plasma DNA levels of EOPD patients were compared with those of healthy controls. Second, the WM microstructural differences between EOPD patients and healthy controls were associated with the differences in cognitive statuses and plasma DNA levels.

## Materials and methods

### Participants

Twenty-four patients (9 males and 15 females; mean age: 48.4 ± 6.5 years) younger than 55 years old with idiopathic PD diagnosed according to the United Kingdom Brain Bank criteria (Gibb and Lees, 1988) and without other neurological disorders or psychiatric disease were prospectively enrolled at the Neurology Department of Chang Gung Memorial Hospital. Each patient's disease severity and functional status were evaluated using the Unified Parkinson's Disease Rating Scale (UPDRS), the modified Hoehn and Yahr Staging Scale, and the Schwab and England Activities of Daily Living Scale. The UPDRS score is the scale most commonly used to follow the longitudinal course of PD and is evaluated by interview and clinical observation (Ramaker et al., 2002). The modified Hoehn and Yahr Scale is a global assessment of the severity of PD based on clinical findings and functional disability, with stages 1 through 5 indicating increasing levels of disease severity (Zhao et al., 2010). The Schwab and England Activities of Daily Living Scale assess the independence of PD patients, with a score of 100% indicating completely independent function while a score of 0% indicates bedridden status with only vegetative function (McRae et al., 2002). Thirty-three sex- and age-matched healthy subjects (15 males and 18 females; mean age: 48.6 ± 7.8 years) with similar education levels and without neurologic disease, psychiatric illness, alcohol, or substance abuse, or head injury were recruited from the hospital as a control group. Participants with cancer, end-stage renal disease, active infection, a history of major trauma, autoimmune disease, or any chronic inflammatory disorder were excluded from both the patient and control groups. The hospital's Institutional Review Committee on Human Research approved the study protocol, and all of the participants or their guardians provided written informed consent.

### Neuro-psychological testing

A clinical psychologist blinded to each patient's status performed a neuro-psychological battery of tests focusing on the attention, executive, speech, and language, memory, and visuospatial functions. Attention functions were assessed by the digit span score from the Wechsler Adult Intelligence scale-III (WAIS-III) (Kaufman and Lichtenberger, 2006) and by the attention and orientation score from the Cognitive Ability Screening Instrument (CASI) (Teng et al., 1994). Executive functions were assessed using the digit symbol coding, similarity, arithmetic, picture arrangement, and matrix reasoning scores from the WAIS-III, as well as the abstract thinking scores from the CASI.

Memory functions were assessed using the short- and long-term memory scores from the CASI and the information scores from the WAIS-III. Speech and language ability were assessed using the vocabulary and comprehension scores from the WAIS-III and the language score from the CASI. Visuospatial functions were assessed using the picture completion and block design scores from the WAIS-III and the drawing score from the CASI.

### Diffusion tensor imaging

#### Image acquisition

The MR data were acquired on a 3.0T whole body GE Signa MRI system (General Electric Healthcare, Milwaukee, WI, USA). For each subject, the subject's head was immobilized with foam pillows inside the coil to diminish motion artifacts. First, the T1-weighted structured images were acquired for localization of FA differences using a 3D-FSPGR sequence (repetition time (TR) = 9.492 ms, echo time (TE) = 3.888 ms, flip angle = 20°, field of view (FOV) = 24 × 24 cm, matrix size = 512 × 512, 110 continuous slices with a slice thickness of 1.3 mm and in-plane spatial resolution of 0.47 × 0.47 mm) through the whole head parallel to the anterior-posterior commissure (AC-PC).

Then, DTI scans were acquired for whole-brain voxel-wise analysis of the WM microstructure using a single-shot echo-planar imaging sequence (TR = 15,800 ms, TE = 77 ms, number of excitations (NEX) = 3, matrix size = 128 × 128, field of view (FOV) = 25.6 cm, voxel size = 2 × 2 × 2.5 mm3, 55 axial oblique slices without gaps). The DTI gradient encoding schemes included 13 non-collinear directions with a *b* value of 1000 s/mm^2^ and a non-diffusion weighted image volume with a *b*-value of 0 s/mm^2^ (null image).

#### Data pre-processing

The FA maps for each subject were computed using an in-house program registered to the ICBM 152 template (Montreal Neurological Institute). In the first step, a group template was created by spatially normalizing each T1-weighted structural MR image to the ICBM 152 template using the optimum 12-parameter affine transformation. These images were subsequently averaged and smoothed with an isotropic 8 mm full-width at half maximum Gaussian kernel.

In the second step, non-diffusion weighted (*b* = 0) images of an individual subject were co-registered to their T1-weighted structural images as the cost function based on normalized mutual information. Thereafter, the registration parameters were applied on the FA maps that were inherently registered to other diffusion-weighted images during the acquisition. To remove non-brain tissue and background noise for these FA maps, the Brain Extraction Tool (BET) compiled in the FSL library 4.1 (Oxford Centre for Functional Magnetic Resonance Imaging of the Brain, Oxford University, Oxford, UK) was applied.

In the third step, an affine transformation was applied with a series of non-linear warps characterized by a linear combination of three dimension discrete cosine transform (DCT) basis functions in order to transform all of the 64 T1-weighted scans to the same stereotactic space as the customized template image. The transformation parameters derived from this step were also applied to the FA maps, which were then effectively registered to the MNI space.

### Laboratory measurement of plasma DNA in the peripheral circulation

Plasma levels of nuclear and mitochondrial DNA were assessed in both the patient and control groups. Blood was drawn by venipuncture from the forearm on the same day as the MRI study and the neuro-psychological testing.

In every patient, 3 ml of peripheral venous blood was collected into ethylenediaminetetraacetic acid–containing tubes. The blood samples were centrifuged for 10 min at 3000 rpm and then transferred to another 1.5 ml polypropylene tube for another centrifugation for 10 min at 10,000 rpm. The plasma samples were frozen at −20°C prior to extraction. The DNA was then extracted from the plasma samples using a QIAamp Blood Kit (Qiagen, Hilden, Germany) according to the manufacturer's protocol.

The plasma DNA was measured by a real-time quantitative polymerase chain reaction (RT-PCR) assay (Roche LightCycler, Roche, Grenzach-Wyhlen, Germany) for the β-globin gene (present in all nucleated cells) and ND2 genes (specific for mitochondrial DNA). Quantitative results were expressed as ng/ml. Procedural details were described in a previous study (Wang et al., 2013).

### Statistical analysis

#### Analysis of demographic data

The demographic data, including the age and sex data, were compared among the study groups using the 2-sample Student *t*-test and Pearson chi-square test, where appropriate, and were reported as mean ± the standard deviation (SD). The significance of differences in Mini-Mental State Examination (MMSE), disease severity, and neuropsychological test scores were analyzed by analysis of covariance (ANCOVA), with the participant's age, sex, and education level as covariates. The plasma DNA levels were compared by Mann-Whitney *U*-test.

#### Group comparison of FA values between patients and controls

Voxel-wise group comparisons were conducted using the SPM8 (Statistical Parametric Mapping; http://www.fil.ion.ucl.ac.uk/spm/; University College London, London, UK) software package in Matlab R2010a (Mathworks, Natick, MA, USA). After normalization and smoothing of the FA images, analysis was performed with SPM8 using the general linear model. ANCOVA was then performed with age and sex as covariates to detect FA differences between the groups. The probability threshold was set at 0.2 to exclude voxels with a substantial probability of containing gray matter or cerebrospinal fluid. The statistical threshold was set at an uncorrected *P* < 0.005, with a cluster of >100 contiguous voxels.

The FSL atlas tool (http://www.fmrib.ox.ac.uk/fsl/fslwiki/Atlases) was applied for determining the most probable fiber tracts and anatomic location of each significant cluster.

Region of interest (ROI) analyses were used to determine the mean *FA* value of each significantly different cluster. The ROI masks were extracted using the Marsbar toolbox (http://marsbar.sourceforge.net/download.html). The mean diffusivity values of these areas were compared between groups by multivariate analysis of covariance, with age and sex as covariates. Significance was set at a Bonferroni corrected *P* < 0.05.

#### Correlations among disease duration, neuro-psychological assessment scores, DTI-related indices, and plasma DNA levels

Partial correlation analysis was performed with age and sex adjustments to determine the associations between DTI changes and plasma DNA levels and disease duration. Partial correlation analysis for assessment of the associations between DTI changes and cognitive functions was performed after controlling for age, sex, and education. The threshold for all statistical significance was set at *P* < 0.05. SPSS software (SPSS V.17, Chicago, IL, USA) was used to perform all the statistical analyses.

## Results

### Baseline clinical characteristics of EOPD patients and controls

**Figure 1:**
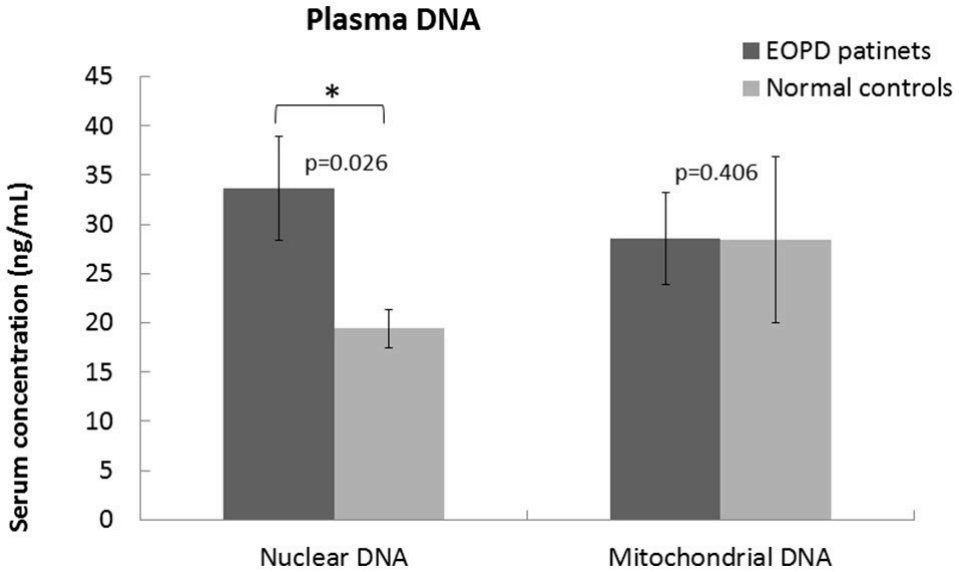
**Quantitative analysis of plasma nuclear and mitochondrial DNA levels in an EOPD patient group (*n* = 24) in comparison with a healthy control group (*n* = 33)**. The plasma nuclear DNA levels were significantly increased (*P* < 0.05) in the EOPD patients; the plasma mitochondrial DNA levels showed no significant difference.

The baseline clinical demographics and neuro-psychological assessment scores of all the subjects are listed in Table 1. Statistical analysis of the clinical demographics showed no significant difference between the two groups. The patients with EOPD performed poorly on the neuro-psychological assessments of executive function [digit symbol coding (*p* = 0.001), arithmetic (*p* = 0.001), matrix reasoning (*p* = 0.028)] and visuospatial function [picture completion (*p* = 0.031), block design (*p* = 0.001), drawing (*p* = 0.007)]. Higher plasma nuclear DNA levels were also observed in the EOPD patients (Figure 1).

**Table 1:** **Demographic data and neuro-psychological assessment data of patients with EOPD and controls**.

**Clinical demographics**	**PD (*n* = 24)**	**Control (*n* = 33)**	***P-value***
Age (year)	48.4 ± 6.5	48.6 ± 7.8	0.911
Sex (M, F)	9, 15	15, 18	0.548
Disease duration (year)	3.23 ± 3.05		
UPDRS I	3.00 ± 2.54		
UPDRS II	7.52 ± 5.85		
UPDRS III	19.09 ± 13.63		
UPDRS total	29.52 ± 20.66		
Modified H & Y	1.74 ± 1.00		
S & E	88.26 ± 13.37		
MMSE	27.38 ± 2.37	28.26 ± 1.83	0.096
**NEURO-PSYCHOLOGICAL ASSESSMENTS**			
**Attention Function**			
Digit span	10.35 ± 3.34	11.22 ± 2.24	0.258
Attention	7.70 ± 0.56	7.44 ± 0.91	0.168
Orientation	17.48 ± 1.56	17.78 ± 0.71	0.386
**Executive Function**			
Digit symbol coding	8.26 ± 3.00	10.56 ± 2.40	0.001^*^
Similarity	9.74 ± 3.37	9.94 ± 2.03	0.726
Arithmetic	8.22 ± 2.52	10.69 ± 2.06	0.001^*^
Picture arrangement	9.44 ± 2.64	10.50 ± 2.48	0.189
Matrix reasoning	8.83 ± 3.68	10.91 ± 2.82	0.028^*^
Abstract thinking	10.09 ± 1.65	10.16 ± 1.39	0.821
**Memory Function**			
Short-term memory	10.47 ± 1.80	10.04 ± 2.04	0.268
Long-term memory	9.91 ± 0.42	10.00 ± 0.00	0.330
Information	9.09 ± 2.31	9.81 ± 2.56	0.523
**Speech and Language**			
Vocabulary	9.70 ± 3.59	10.63 ± 3.02	0.057
Comprehension	9.30 ± 3.01	9.78 ± 3.05	0.912
Language	9.79 ± 0.42	9.88 ± 0.44	0.648
**Visuospatial Function**			
Picture completion	8.48 ± 2.83	10.72 ± 3.74	0.031^*^
Block design	8.22 ± 2.73	10.53 ± 2.38	0.001^*^
Drawing	9.44 ± 1.08	10.00 ± 0.00	0.007^*^

*UPDRS, Unified Parkinson's Disease Rating Scale; Modified H & Y, Modified Hoehn and Yahr stages; S & E, Schwab and England activities of daily living scale; MMSE, Mini Mental State Examination*.

*Sex data were compared by Pearson chi-square test. Age data were compared by independent t-test. MMSE and neuro-psychological assessment data were compared by analysis of covariance (ANCOVA) after controlling for age, sex, and education*.

Data are presented as mean ± standard deviation.

* *p < 0.05*.

### Group comparisons on FA maps

**Figure 2:**
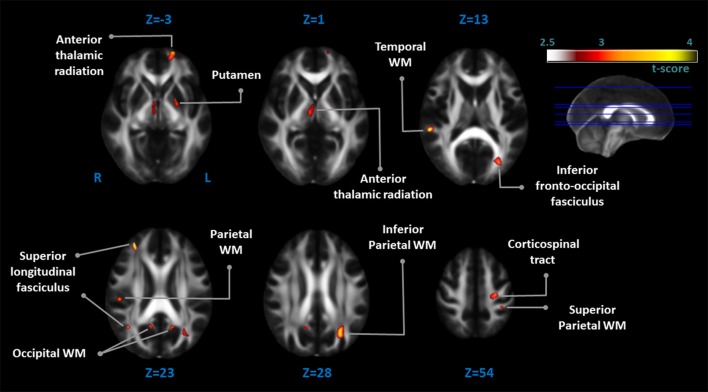
**Comparison of fractional anisotropy *(FA)* values between EOPD patients and healthy controls**. There were diffuse diffusion tensor imaging (DTI) deficits in the EOPD group as compared with the healthy control group. Orange voxels indicated regions with significantly lower *FA* values in the EOPD patients vs. the healthy controls (*p* < 0.005, uncorrected).

The EOPD patients had significantly lower *FA* values in several WM regions than the control group. Together with these lower *FA* values, there were WM regions with increased mean diffusivity (MD) values in the left inferior parietal WM, bilateral occipital WM, right parietal WM, and right posterior superior longitudinal fasciculus (Table 2; Figure 2).

**Table 2:** **Regions showing fractional anisotropy differences among patients with PD and control subjects**.

**MNI atlas coordinates**						***FA*****, mean (SD)**			**Diffusivity Values (PD-NC) (x10^−6^mm2/s)**
**x**	**y**	**z**	**Voxel size**	**White matter tract**	**Nearest cortical area**	**Controls**	**PD**	**T_max_**	**MD**
**DECREASED FA IN PD vs. CONTROLS**									
30	38	21	320	Right anterior superior longitudinal fasciculus	Right frontal lobe	0.294 (0.049)	0.263 (0.058)	4.19	−0.394
55	−34	13	293	Right temporal WM	Right temporal lobe	0.348 (0.038)	0.310 (0.036)	4	−6.277
−27	−63	28	920	Left inferior parietal WM	Left parietal lobe	0.424 (0.040)	0.386 (0.027)	3.82	34.348^*^
−18	59	−3	435	Left anterior thalamic radiation	Left frontal lobe	0.329 (0.033)	0.305 (0.019)	3.61	17.511
−15	−59	24	140	Left occipital WM	Left occipital lobe	0.298 (0.048)	0.260 (0.038)	3.53	28.667^*^
49	−25	23	109	Right pariental WM	Right parietal lobe	0.271 (0.047)	0.231 (0.066)	3.39	71.144^*^
39	−58	23	100	Right posterior superior longitudinal fasciculus	Right temporal lobe	0.369 (0.065)	0.314 (0.048)	3.39	39.292^*^
−29	−75	14	422	Left inferior fronto-occipital fasciculus	Left occipital lobe	0.602 (0.046)	0.562 (0.038)	3.35	23.057
−23	−19	54	575	Left corticospinal tract	Left frontal lobe	0.569 (0.037)	0.539 (0.038)	3.29	14.837
13	−58	24	252	Right occipital WM	Right occipital Lobe	0.327 (0.046)	0.286 (0.050)	3.28	59.337^*^
−22	0	−3	219	Left putamen	Left lentiform nucleus	0.252 (0.031)	0.229 (0.016)	3.27	1.852
7	−13	1	841	Right anterior thalamic radiation	Right thalamus	0.334 (0.019)	0.316 (0.018)	3.08	26.606
−31	−34	54	122	Left superior pariental WM	Left parietal lobe	0.564 (0.053)	0.491 (0.060)	2.99	25.898

*Location of maximum effect (uncorrected P < 0.005, cluster size > 100) was shown in the Montreal Neurological Institute (MNI) space. Group FA mean values in each cluster are presented as mean ± standard deviation*.

The FA and MD values in region of interest were further compared between two groups by analysis of covariance after controlling for age and sex.

* *P < 0.05 with a Bonferroni correction, accounting for multiple ROI comparisons. WM, white matter; FA, fractional anisotropy; MD, mean diffusivity; PD, Parkinson's disease; NC, Normal Controls*.

### Correlations between disease duration and regional DTI-related indices and plasma DNA levels

There were no significant correlations between disease duration and DTI signal changes or plasma DNA levels.

### Correlations between regional DTI-related indices and neuro-psychological assessment scores

There were significant correlations between worse visuospatial function (drawing) scores and increased *MD* values of the left anterior thalamic radiation and decreased *FA* values of the left superior parietal WM. Furthermore, worse executive function (arithmetic) was associated with increased *MD* values of the right anterior thalamic radiation (Table 3).

**Table 3:** **Correlations among diffusion tensor imaging (DTI) abnormalities, cognitive function, and plasma DNA levels**.

**Anatomy**	**DTI**	**Poor NPT (r,p)**	**↑Plasma DNA(r,p)**
Left anterior thalamic radiation	↑MD	Drawing (−0.857, 0.015)	
Left occipital WM	↑MD		Nuclear DNA (0.307, 0.028)
Right parietal WM	↑MD		Nuclear DNA (0.352, 0.011)
Left corticospinal Tract	↓FA		Nuclear DNA (−0.284, 0.043)
Right occipital WM	↑MD		Nuclear DNA (0.343, 0.014)
Right anterior thalamic radiation	↓FA		Nuclear DNA (−0.349, 0.012)
	↑MD	Arithmetic (−0.878, 0.009)	
Left superior parietal WM	↓FA	Drawing (0.780, 0.038)	

Correlations among diffusion tensor abnormalities and neuro-psychological test (NPT) scores were performed by partial correlation after controlling for age, sex, and education. Correlations between diffusion tensor abnormalities and plasma DNA levels were performed by partial correlation after controlling for age and sex. * P < 0.05 with a Bonferroni correction, accounting for multiple region of interest comparisons. FA, fractional anisotropy; MD, mean diffusivity; WM, white matter.

### Correlations between regional DTI-related indices and plasma DNA levels

There were significant correlations between increased nuclear DNA levels and decreased *FA* values of the left corticospinal tract and right anterior thalamic radiation, as well as increased *MD* values of the bilateral occipital WM and right parietal WM (Table 3).

## Discussion

In this study, we found that EOPD patients have higher plasma nuclear DNA levels and greater cognitive impairments in the executive and visuospatial function domains than healthy individuals, as well as extensive WM microstructural changes as indicated by decreased FA or increased *MD* values. These WM microstructural changes were associated with elevated plasma DNA levels and cognitive impairments in the executive and visuospatial domains.

PD may cause more substantial psychosocial and socioeconomic impacts on younger patients. They may experience early unemployment, poor quality of life, depression, and marital discord (Schrag et al., 2003). Furthermore, the disease also places burdens on a patient's spouse and children (Carter et al., 1998; Schrag et al., 2004). In this study, we focused on relatively young PD patients who experienced disease onset before 55 years of age. Although this cutoff of 55 years of age is somewhat higher than that traditionally used to define EOPD, our patients, with a mean age of <50 years old, were still relatively young in comparison to patients with regular PD, such that many are still working and may also have young children to raise. As such, the motor symptoms and cognitive impairment resulting from their EOPD may cause greater impacts on these patients.

The cognitive impairment seen in PD patients is well-documented. PD patients exhibit frontal-executive cognitive impairments similar to those of patients with frontal lobe lesions (Kehagia et al., 2010). While the cognitive impairments in PD patients may involve the attention, executive, memory, visuospatial, and language domains, the most commonly impaired domain is memory (Aarsland et al., 2010). The cognitive impairment seen in EOPD patients is less well-studied. Among the available studies, some have reported no significant cognitive impairments in EOPD patients (Schrag and Schott, 2006; Wickremaratchi et al., 2009; Caccappolo et al., 2011), while others have reported that EOPD patients may have some cognitive impairments (Hietanen and Teräväinen, 1988; Dubois et al., 1990; Tsai et al., 1994). The discrepancies between the existing studies may be due to differences in the ages and disease severities of the patients investigated, as well as differences in the neuropsychological testing methods applied. In this study, we performed comprehensive neuropsychological assessments of EOPD patients who were in relatively early stages of the disease in terms of disease severity. We found significant cognitive impairments in the executive and visuospatial domains. These results may imply underlying brain structural changes in EOPD patients.

DTI is an advanced MRI technique that can assess brain microstructures noninvasively by measuring both the direction and magnitude of water molecule diffusion. The most commonly used parameters in DTI are FA and MD. FA measures the proportion of directionality of water diffusion and therefore reflects the integrity of the myelination and organization of axon fibers (Laule et al., 2007; Johansen-Berg and Rushworth, 2009). MD measures the quantity of overall diffusion in a given voxel regardless of its direction and therefore reflects the overall density or integrity of WM fibers (Burzynska et al., 2010; Rae et al., 2012).

To the best of our knowledge, we are the first group to report WM microstructural changes using DTI in EOPD patients. Previous DTI studies of PD patients have shown extensive WM microstructural changes, albeit with a diversity of results, with most studies reporting WM changes in the frontal lobe (Cochrane and Ebmeier, 2013; Kim et al., 2013). Similar to the results previously found for PD patients, we found extensive WM microstructural changes, as indicated by a decrease in *FA* values or an increase in *MD* values, involving the frontal, parietal, temporal, and occipital lobes in the EOPD patients. These changes may be related to the pathological mechanisms of EOPD. Among the brain regions with significant DTI changes identified in this study, an isolated decrease in *FA* values was identified mostly in the frontal lobe and nearby deep gray matter, which may reflect underlying demyelination in these brain regions in EOPD patients. Decreased *FA* values along with increased *MD* values were identified mostly in the parietal and occipital lobes, which may reflect gliosis or axonal loss in these brain regions in EOPD patients.

Cognitive function is associated with cortical atrophy in PD patients, such that PD patients with cognitive impairments may show more extensive cortical atrophy and decreased cortical perfusion compared with PD patients without cognitive impairments (Ibarretxe-Bilbao et al., 2009; Chen et al., 2016; Lin et al., 2016) Relatedly, in PD patients with dementia, a previous study reported extensive frontal and subcortical atrophy compared with PD patients with no cognitive impairment (Borroni et al., 2015). The results of this study, meanwhile, show extensive WM changes even in the early stages of the disease. Future studies with voxel-based morphometry analysis in combination with DTI analysis may further clarify the relationship between gray matter atrophy and WM changes in EOPD patients.

Plasma DNA levels can become elevated due to various insults causing cell death with subsequent release of DNA into the circulating plasma. Recent studies have also found increased plasma nuclear DNA levels in chronic neurodegeneration disorders like Friedreich's ataxia and spinocerebellar ataxia (Swarup et al., 2011). In PD, oxidative stress is an important contributing factor in the development of the disease. Elevated oxidative stress, along with neuroinflammation, leads to neural cell damage and death (Hald and Lotharius, 2005; Chen et al., 2015). This neuron death, accompanied by the breakdown of the blood-brain barrier, causes the release of DNA into the plasma. Furthermore, since PD patients have increased systemic inflammation and peripheral cell death that are correlated with disease severity, increased cell death with the release of DNA into the plasma may occur (Lin et al., 2014b, 2015; Macchi et al., 2015; Yu et al., 2016). Either way, the levels of plasma nuclear DNA may rise. In this study, we found increased levels of plasma nuclear DNA in the EOPD patient group. We also found significant associations between elevated plasma nuclear DNA levels and WM microstructural changes. This finding supports the notion that EOPD patients may exhibit increased oxidative stress with possible associated neural damage and death that lead, in turn, to extensive WM microstructural changes visible via DTI. However, further longitudinal studies are needed to delineate the causal relationships between the elevated oxidative stress and WM changes in EOPD patients.

The results of this study also indicated associations between executive function impairment and WM microstructural changes in the anterior thalamic radiation, a finding which is similar to that of a previous study conducted on PD patients (Zheng et al., 2014). This WM region, which is adjacent to the thalamus, is also associated with elevated plasma nuclear DNA levels. According to the Braak staging, pathology in the PD brain spreads from the dorsal nucleus of the vagus nerve (stage I) to the brain stem in an ascending fashion from the medulla (stage II) to the midbrain (stage III) to the mesocortex, basal forebrain, and thalamus (stage IV) and then to the neocortex (stages V and VI) (Braak et al., 2003). It is not until after stage III that patients may start to present with motor symptoms (Dexter and Jenner, 2013). Since the patients in this study were still in the relatively early stages of the disease (with the mean modified Hoehn and Yahr Stage scores of <2), our results implied that the thalamus may be an important region in the pathogenesis of EOPD, and that it is affected early in the disease process. This finding is compatible with the notion that the executive function impairments in PD are due to alterations in the corticostriatal pathway (Albin et al., 1989; Chou et al., 2015). Recently, functional MRI studies have demonstrated that the executive function impairments in PD are mediated not only by alterations in the corticostriatal pathway but also the mesolimbic pathway (Monchi et al., 2004, 2007; Au et al., 2012). Future studies with functional MRI focusing on specific types of executive function assessment may further delineate the complex relationships between the brain activation pathways and executive impairments in EOPD patients.

### Limitations

This study does have several limitations. First, the patients who participated were recruited from a single tertiary center and so may not be representative of all EOPD populations. Second, due to the relatively small sample of patients, the association analysis in this study only included items for which the EOPD patients scored significantly poorer than the control group. Third, this study was a cross-sectional study and, as such, readers should interpret the results with caution. Further causal relationships among DTI changes, cognitive function changes, and plasma DNA levels may thus need to be delineated by future longitudinal studies. Lastly, although DTI indices such as FA and MD are sensitive markers for detecting brain microstructural changes, they lack specificity (Pierpaoli and Basser, 1996). Using newer diffusion MR technologies such as neurite orientation dispersion and density imaging (NODDI), which separates diffusion in the brain into intraneurite, extraneurite, and CSF compartments, may increase the specificity for identifying WM microstructural changes (Zhang et al., 2012).

## Conclusion

In this study we found extensive WM damage and elevated systemic oxidative stress in EOPD patients. Our findings support the notion that thalamus and its adjacent anterior thalamic radiation may be important in the pathogenesis of EOPD, as it appears to become involved in the disease process at an early stage. A longitudinal study may further delineate the causal relationships among the cognitive impairment, DTI changes, and plasma DNA level alterations found in EOPD patients.

## Ethics statement

Institutional Review Board in Chang-Gung Memorial Hospital. Institutional Review Board in Chang-Gung Memorial Hospital approved the study protocol, and all of the participants or their guardians provided written informed consent. All of the participants or their guardians provided written informed consent.

## Author contributions

WL designed the study. YC and MC conducted the statistical analysis. CL participated in the patient enrollment. PC conducted the imaging pre-processing. HC and IY conducted the laboratory analysis. YC drafted the manuscript. NT conducted the neuro-psychological assessment. All authors critically reviewed the manuscript, contributed to its revision, and approved the final version submitted. All authors had full access to all of the data (including statistical reports, tables, and figures) in the study and can take responsibility for the integrity of the data and the accuracy of the data analysis.

## Funding

This was not an industry supported study. This work was supported by funds from the National Science Council (MOST 103-2314-B-182A-010 -MY3 to WL and MOST 104-2314-B-182A-053 to HC). The authors have indicated no financial conflicts of interest.

### Conflict of interest statement

The authors declare that the research was conducted in the absence of any commercial or financial relationships that could be construed as a potential conflict of interest. The reviewer TF and handling Editor declared their shared affiliation, and the handling Editor states that the process nevertheless met the standards of a fair and objective review.

## Abbreviations

WM: white matter
PD: Parkinson's disease
EOPD: early-onset Parkinson's disease
DTI: diffusion tensor imaging
FA: fractional anisotropy
MD: mean diffusivity
UPDRS: Unified Parkinson's Disease Rating Scale
modified H & Y scale: modified Hoehn and Yahr Staging Scale
S & E scale: Schwab and England Activities of Daily Living Scale
ANCOVA: analysis of covariance
WAIS-III: Wechsler Adult Intelligence scale-III
CASI: Cognitive Ability Screening Instrument.
